# Risk Prediction of Cerebrovascular Ischemic Events Following Cervical Artery Dissections Using High‐Intensity Transient Signals: A Systematic Review, Meta‐Analysis and a Single Center Experience

**DOI:** 10.1161/SVIN.124.001704

**Published:** 2025-03-08

**Authors:** Seyed Behnam Jazayeri, Behnam Sabayan, Yasaman Pirahanchi, Vikas Ravi, Julián Carrión‐Penagos, Jeffery Bowers, Royya Modir, Kunal Agrawal, Thomas Hemmen, Brett Meyer, Dawn Meyer, Reza Bavarsad Shahripour

**Affiliations:** ^1^ Sina Trauma and Surgery Research Center Tehran University of Medical Sciences Tehran Iran; ^2^ Department of Neurology Hennepin Healthcare Research Institute Minneapolis MN; ^3^ Division of Epidemiology and Community Health School of Public Health University of Minnesota Minneapolis MN; ^4^ Neuroscience Department Comprehensive Stroke Center University of California San Diego San Diego CA

**Keywords:** dissection, high‐intensity transient signals, ischemic events, microembolic signals, stroke, transcranial doppler

## Abstract

**Background:**

Predicting and managing spontaneous cervical artery dissections (CeAD) is challenging due to the absence of tools for early identification of high‐risk individuals. This study seeks to gather evidence on the predictive value of high‐intensity transient signals (HITS) detected by transcranial Doppler for recurrent ischemic events (IEs) following CeAD.

**Methods:**

We performed a systematic review and meta‐analysis of published studies along with the data from our cohort. Following Preferred Reporting Items for Systematic Reviews and Meta‐Analyses guidelines, we searched PubMed, Embase, and Scopus to identify studies that evaluated HITS in patients with CeAD with the aim of predicting IEs. Data were pooled using a random effects model, with odds ratio (OR) and its 95% CI as the effect size. Heterogeneity was assessed with the Q statistic and I^2^ test, and subgroup analysis evaluated the impact of dissected artery (carotid versus vertebral) on the relationship between HITS and IEs. Our retrospective study included consecutive patients diagnosed with CeAD, followed for 90 days to record IEs. Univariable and multivariable analyses were performed to identify factors associated with recurrent transient ischemic attacks or strokes within 90 days post CeAD.

**Results:**

Our systematic review included 5 prior studies, which, combined with our center's sample size, provided data for a total of 306 patients. The meta‐analysis indicated that HITS is a significant predictor of IEs (OR: 13.25 [95% CI, 2.97–59.13], *P*<0.01) with low heterogeneity (I^2^ = 42%, *P* = 0.13). However, subgroup analysis indicated that HITS are a significant predictor only for carotid artery dissections (*P*<0.01) and not for vertebral artery dissections (*P* = 0.11). The cohort consisted of 34 patients (mean age: 46.8 years, 55.9% male). The incidence of IEs was 20% in our cohort and all of them (100%) had HITSs in transcranial Doppler. In multivariable analysis, the presence of HITS (*P* = 0.006) and intraluminal thrombosis (*P* = 0.02) were significant predictors of IEs.

**Conclusion:**

The presence of HITS detected by transcranial Doppler is a strong predictor of IEs in patients with carotid artery dissections. This highlights the clinical value of transcranial Doppler in identifying high‐risk patients and emphasizes the need for proactive management strategies to reduce the risk of future IEs in this subgroup.

## Graphical abstract

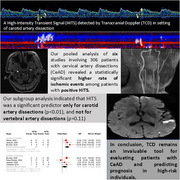

**Nonstandard Abbreviations and Acronyms**

CAD: carotid artery dissection
CeAD: cervical artery dissection
HITS: high‐intensity transient signals
IE: ischemic event
TCD: Transcranial Doppler
VAD: vertebral artery dissection

**Clinical Perspective**

**What Is New?**

- This study provides the first systematic review and meta‐analysis evaluating the predictive value of high‐intensity transient signals detected by transcranial Doppler for recurrent ischemic events in patients with spontaneous cervical artery dissection.
- It identifies that high‐intensity transient signals is a significant predictor of ischemic events specifically in carotid artery dissections but not in vertebral artery dissections, refining the risk stratification for these patients.

**What Are the Clinical Implications?**

- Transcranial Doppler could serve as a valuable non‐invasive tool for early identification of high‐risk cervical artery dissection patients, allowing for more targeted surveillance and timely intervention.
- Patients with carotid artery dissections and detected high‐intensity transient signals may benefit from closer monitoring and potentially more aggressive management strategies to reduce the risk of recurrent ischemic events.

Internal carotid and vertebral artery dissections, collectively referred to as cervical arterial dissection (CeAD), are key mechanisms for ischemic stroke in young adults and account for approximately 25% of strokes in patients 50 years and younger.^1^ Predicting the risk of complications, such as ischemic stroke, in patients with CeAD presents several challenges due to the lack of reliable tools for early identification of high‐risk individuals. Another key issue is the absence of a standardized approach for managing CeAD with antithrombotic therapies. Deciding on the optimal treatment—whether anticoagulation, single antiplatelet therapy, or dual antiplatelet therapy—requires careful consideration of patient‐specific factors, particularly the risk of distal embolic events.

Transcranial Doppler (TCD), a noninvasive bedside imaging technique, can play a pivotal role as a risk biomarker by allowing clinicians to monitor blood flow, quantify blood flow velocity, and, most importantly, detect microembolic particles in real time using high‐intensity transient signals (HITS) within the intracranial vessels.^2^, ^3^ Given this capability, TCD has been validated as a key tool to identify individuals at high risk for future cerebrovascular events in the setting of asymptomatic carotid stenosis.^4^

Several studies have investigated the prevalence of HITS in patients with CeAD and explored their potential to predict ischemic events (IEs) in this population. In the present study, we systematically reviewed and analyzed previous research to evaluate the predictive value of TCD‐detected HITS in determining the likelihood of IEs following CeAD. In addition, we report our center's experience in detecting HITS and other predictors of IEs following CeAD.

## Methods

The data that support the findings of this study are available from the corresponding author upon reasonable request.

### Search Strategy and Study Selection Criteria

This study adhered to the guidelines set by the Preferred Reporting Items for Systematic Reviews and Meta‐Analyses.^5^ The Preferred Reporting Items for Systematic Reviews and Meta‐Analyses checklist is provided in supplementary materials as Table S1. The protocol of this review was not registered. We conducted a comprehensive search of PubMed, Embase, and Scopus databases to identify studies that evaluated HITS in patients with CeAD with the aim of predicting IEs. The search keywords included “transcranial Doppler,” “TCD,” “TCCS,” “stroke,” “TIA,” “ischemic event,” “vertebral artery dissection,” “carotid artery dissection,” “HITS,” and “microembolic signals.” The full search syntax for each database is provided in Table S2. The search was limited to English‐language articles from inception to September 2024. Additional studies were identified by screening reference lists of relevant articles. Two reviewers independently screened the titles and abstracts of identified studies. Full‐text articles of potentially relevant studies were retrieved and assessed for eligibility. We excluded case reports, case series with <5 cases, letters, correspondence, reviews, editorials, conference abstracts, and nonhuman articles. Data extracted included study design, sample size, patient demographics, time since onset of symptoms to TCD evaluation, antiplatelet/anticoagulation treatment before TCD, HITS, and IEs at follow‐up. Data were extracted by a single author and confirmed with another author. Discrepancies were resolved through discussion or consultation with a third reviewer.

### Quality Appraisal

We used the Newcastle‐Ottawa scale^6^ to assess the quality of cohort studies, and used the Murad et al's tool^7^ for assessing the quality of case series. Two reviewers assessed the quality of studies and a third author was consulted in case of any discrepancy. For the Newcastle‐Ottawa scale assessment, total scores were classified into 3 categories: (1) 1–3 indicating a high risk of bias; (2) 4–6 suggesting some concerns; and (3) 7–9 representing a low risk of bias. The case series quality assessment tool included questions across 4 domains: selection bias, ascertainment bias, causality, and reporting bias. Studies were categorized based on their total scores into high risk (1–3 points), some concern (4 points), and low risk (5 points).

### Statistical Analysis

Meta‐analyses were conducted using R software version 4.3.2 (R Project for Statistical Computing) meta package version 6.5–0. We calculated odds ratios (ORs) and their corresponding 95% CIs using a random‐effects model (generalized linear mixed models) to account for methodological heterogeneities.^8^ Heterogeneity was assessed using Q statistic and I^2^ test, with I^2^ >50% or *P*<0.05 considered significant. Because no heterogeneity was observed during the analysis, a sensitivity analysis was not conducted. Additionally, as fewer than 10 studies were included in the analysis, it was not feasible to perform the Egger's test for publication bias or to conduct a meta‐regression.^9^, ^10^ To assess the impact of the dissected artery (carotid versus vertebral) on the relationship between HITS and IEs, a subgroup analysis was performed.

Details of methods for our cohort study is provided in supplementary materials. In short, we followed the Strengthening the Reporting of Observational Studies in Epidemiology (STROBE) guideline for observational studies. The STROBE checklist is provided in Table S3. We conducted a retrospective study involving all consecutive patients with CeAD who were admitted to the University of California San Diego Medical Center between February 2021 and December 2023. The inclusion criteria for this study were as follows: (1) carotid or vertebral artery dissection confirmed by advanced imaging, (2) age ≥18 years, and (3) TCD study during the first 72 hours from symptom onset. We did not enroll patients with (1) poor TCD window, (2) traumatic dissections, and (3) TCD evaluation after 72 hours from symptoms. Univariable and multivariable analyses were performed to identify factors associated with IEs following CeAD. Potential factors of IE were identified based on Yaghi et al's study.^11^

## Results

**Figure 1:**
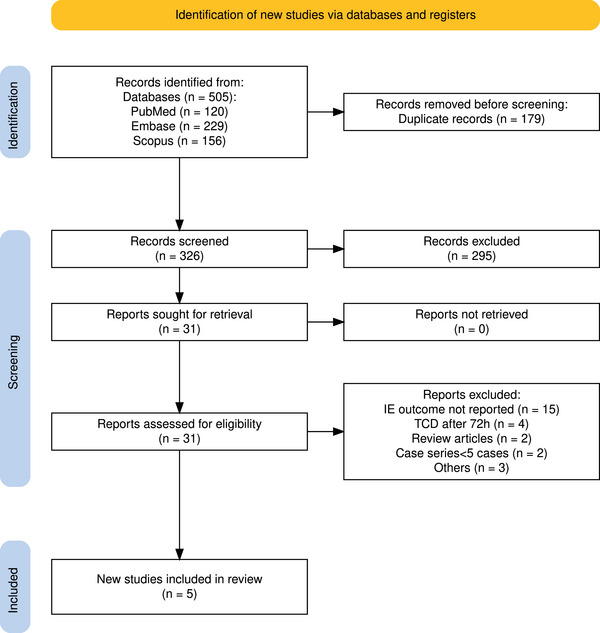
**Preferred Reporting Items for Systematic Reviews and Meta‐Analyses flow chart of study selection**. IE indicates ischemic event; and TCD, transcranial Doppler.

PubMed, Embase, and Scopus database search yielded a total of 505 articles. After the duplicates (179 articles) were removed, the title and abstract of the remaining articles were scanned, and full texts of 326 studies were screened. A total of 5 reports were selected based on the inclusion and exclusion criteria (Figure 1).^12^, ^13^, ^14^, ^15^, ^16^ Two studies were excluded at full‐text stage because they had <5 cases.

The studies, including our cohort, provided data on 306 patients with CeAD who were monitored for IEs and evaluated by TCD to assess the predictive value of HITS. The analysis included 3 prospective cohorts,^12^, ^13^, ^16^ 2 retrospective cohorts,^14^ including our center's experience, and 1 case series.^15^ Overall, 4 studies had low risk of bias, and 2 studies had some concerns. The detailed risk of bias assessment is presented in Tables S4 and S5. The average age of patients was 45.0 (5.8) years and 52% were male. Carotid artery dissection was present in 97 patients and vertebral arteries in 209 patients. A summary of study characteristics is presented in Table 1.

**Table 1:** Summary of Study Characteristics from Literature

Study	Period of observation	Country	Design	Age, y (mean±SD), %male	Sample size, n (%)	Dissection artery, n (%)	Type of dissection, n (%)	Onset to TCD evaluation	Radiographic modality to diagnose dissection, n (%)	Antiplatelet or anticoagulant therapy before TCD examination, n (%)	Comorbidities	Follow‐up duration	IEs at follow‐up based on HITS status, n/N (%)
Present study	February 2021–december 2023	United States	Retrospective cohort	46.8 ± 11.2, 55.9%	HITS+16 (47.0) HITS‐18 (53.0) Total: 34	Carotid: 28 (82.3) Vertebral: 6 (17.7)	Spontaneous: 34 (100)	<24 h: 24 (70.6) 24–48 h: 9 (26.5) 48–72 h: 1 (2.9)	CTA: 34 (100) MRA: 14 (41.2) Angiography: 9 (26.5)	Before TCD exam: SAPT (ASA): 5 (14.7) DAPT: 2 (5.9) anticoagulant: 1 (2.9)	hypertension: 9 (26.5) DM: 4 (11.8) hyperlipidemia: 4 (11.8) Smoking: 10 (29.4) Migraine: 9 (26.5)	90 d	HITS+7/16 (43.7) HITS‐ 0/18 (0) Total: 7/34 (20.6)
Perez‐Roman, 2024	January 2009–January 2018	United States	Retrospective cohort	53.2 ± 15.4, 70.1%	HITS+17 (16.4) HITS‐87 (83.6) Total: 104	Vertebral: 104 (100)	Spontaneous: 60 (57.7) Traumatic: 40 (38.5) Iatrogenic: 4 (3.8)	‐	CTA: 21 (20.2) MRA: 49 (47.1) Angiography: 34 (32.7)	On the same day of TCD: SAPT: 47 (45.2) DAPT: 25 (24.0) anticoagulant: 9 (27.9)	‐	‐	HITS+ 9/17 (52.9) HITS‐ 51/87 (58.6) Total: 60/104 (56.6)
Brunser, 2020	June 2017–February 2020	Chile	Prospective cohort	37.9 ± 7.5, 14.8%	HITS+4 (4.5) HITS‐84 (95.5) Total: 88	Vertebral: 88 (100)	Spontaneous: 88 (100)	4.89 ± 5 d	CTA and MRA: 42 (47.7) CTA and angiography: 4 (4.4)	SAPT: ASA 100 mg at admission 88 (100) DAPT: ‐ anticoagulant: ‐	hypertension: 6 (6.8) DM: 3 (3.4%) hyperlipidemia: 8 (9%) Smoking: 13 (14.7) Migraine: 37 (42)	90 d	HITS+ 2/4 (50.0) HITS‐ 4/84 (4.7) Total: 6/88 (6.8)
Brunser, 2016	April 2004–January 2015	Chile	Prospective cohort	41.9 (range: 18–56) 75.6%	HITS+3 (7.3) HITS‐38 (92.7) Total: 41	Carotid: 41 (100) 45 carotid and 11 vertebral artery dissections in 41 patients	Spontaneous: 41 (100)	4.1 ± 3.5 d	CTA and MRA: 11 (26.8) CTA and angiography: 15 (36.5)	SAPT: 17 (41.4) DAPT: ‐ anticoagulant: 24 (58.6)	hypertension: 10 (24.3) DM: 3 1 (2.4) hyperlipidemia: 6 (14.6) Smoking: 5 (12.1)	90 d	HITS+ 3/4 (75.0) HITS‐ 0/37 (0) Total: 3/41 (7.3)
Yamaoka, 2014	‐	Japan	Case series	45.5, 81.2%	HITS+2 (17.8) HITS‐9 (82.2) Total 11	Vertebral: 11 (100)	Spontaneous: 9 (82.2) Traumatic: 2 (17.8)	Within 72 h	MRI, MRA or angiography	SAPT: 2 (18.2) DAPT: ‐ anticoagulant: 2 (18.2)	‐	‐	HITS+ 1/2 (50) HITS‐ 0/9 (0) Total: 1/11 (9.0)
Molina, 2000	September 1997–February 2000	Spain	Prospective cohort	41.5 (range 21–64), 71.4%	HITS+13 (46.4) HITS‐15 (53.6) Total 28	Carotid: 28 (100)	Spontaneous: 28 (100)	< 24 h and repeated for 3 times on subsequent days	MRI ± angiography	None After first TCD: All received heparin.	‐	‐	HITS+ 6/13 (46.1) HITS‐ 1/15 (6.7) Total: 7/28 (25)

ASA indicates acetylsalicylic acid; CTA, computed tomography angiography; DAPT, dual antiplatelet therapy; DM, diabetes mellitus; HITS, high‐intensity transient signals; IEs, ischemic events; MRA, magnetic resonance angiography; MRI, magnetic resonance imaging; SAPT, single antiplatelet therapy; and TCD, transcranial Doppler.

**Figure 2:**
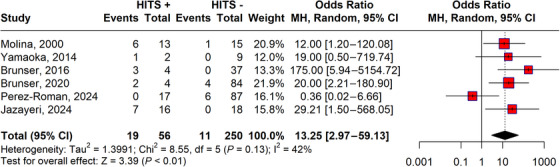
**Forest plot of ischemic events following CeAD based on presence or absence of HITS**. CeAD indicates cervical artery dissections; HITS, high‐intensity transient signals; IEs, ischemic events; and MH, Mantel–Haenszel.

**Figure 3:**
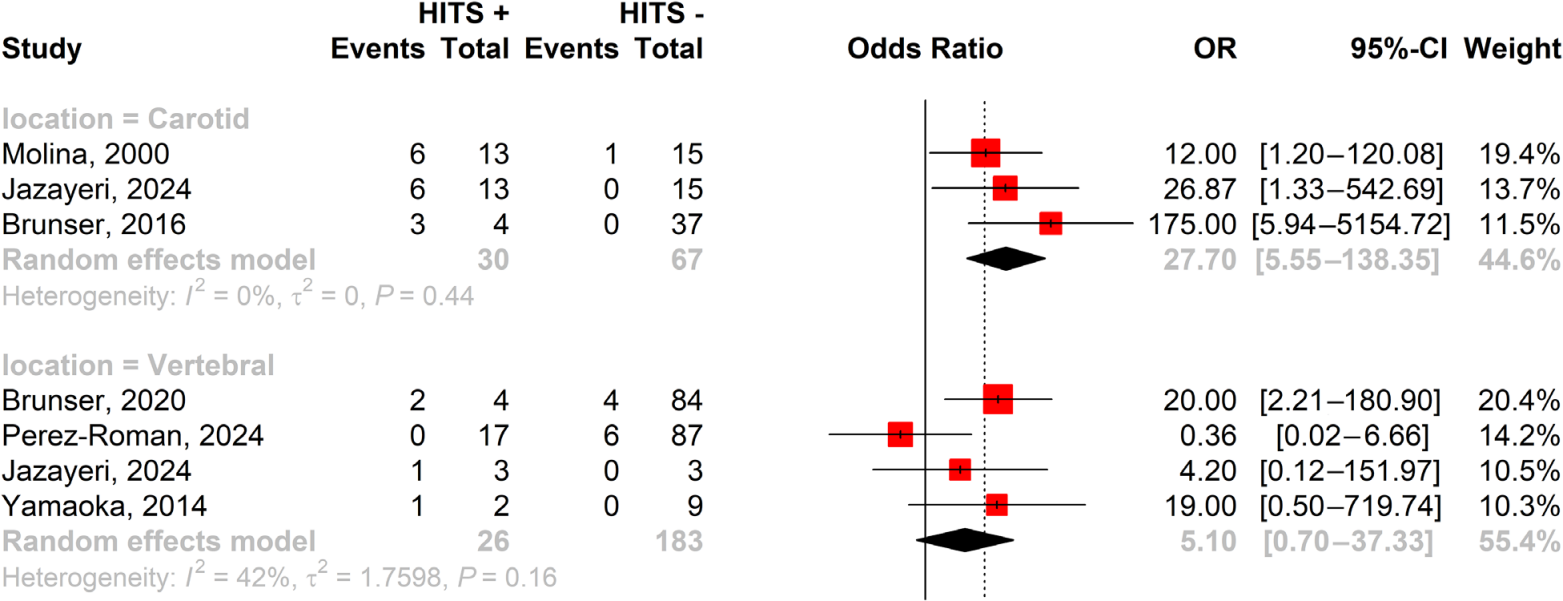
**Subgroup analysis of the results based on dissected artery**. HITS indicates high‐intensity transient signals; and OR, odds ratio.

Pooled analysis of 6 studies involving 306 patients revealed a statistically significant higher rate of IEs among patients with positive HITS (OR, 13.25 [95% CI, 2.97–59.13], *P*<0.01), with low heterogeneity across the studies (I^2^ = 42%, *P* = 0.13) (Figure 2). However, our subgroup analysis indicated that HITS was a significant predictor only for carotid artery dissections (CAD) (OR, 27.7 [95% CI, 5.55–138.35]) and not for vertebral artery dissections (OR, 5.10 [95% CI, 0.7–37.7]) (Figure 3).

Details of our single‐center cohort are provided in supplementary Tables S6, S7 and S8. The mean age of 34 included patients was 46.8±11.2 years, and 56% were male. In total, HITS were observed in 16 patients (47%). Patients with HITS had similar age, gender, dissected artery, comorbidities, and luminal thrombosis to patients without HITS (Table S8). However, luminal stenosis was higher in patients with HITS (*P* = 0.008). Among 16 patients with HITS, 7 patients experienced IEs (43.7%) compared with no IEs among 18 patients without HITS. The difference was statistically significant (*P* = 0.002). In the univariable analysis, high‐grade luminal stenosis, luminal thrombosis, and the presence of HITS were significantly associated with recurrent IEs. In the multivariable analysis, the presence of HITS (OR, 29.7 [95% CI, 2.5–341.7, *P* = 0.006) and luminal thrombosis (OR, 20 [95% CI, 1.4–282, *P* = 0.02) remained significant predictors of IEs, after adjusting for the degree of luminal stenosis (Table S7). Further details of study patient characteristics and outcomes are provided in supplemental materials.

## Discussion

CeAD is a relatively rare cause of ischemic strokes overall, accounting for only 1%–2% of all cases. However, it plays a much more significant role in younger adults, where it is responsible for 10%–25% of ischemic strokes.^1^, ^17^ Although hypoperfusion can contribute to ischemia, research indicates that thromboembolism is the primary cause of most ischemic strokes in patients with CeAD.^18^ Therefore, early diagnosis of thromboembolism and appropriate intervention strategies, such as antithrombotic therapy, are essential. Regular follow‐up using techniques like TCD to monitor for HITS can aid in identifying patients at higher risk of recurrent IEs. Previously, Sudheer and Vibha conducted a meta‐analysis of 112 patients, demonstrating that HITS occurred in approximately 46% of patients with CeAD. In addition, the presence of microembolic signals was linked to an increased risk of early ischemic recurrence in patients with CeAD.^19^ Our analysis, which includes a larger number of studies, provides additional evidence supporting the prognostic value of HITS with enhanced statistical power. Furthermore, our subgroup analysis highlighted the distinct predictive value of HITS in both carotid and vertebral artery dissections, offering more detailed insights into its role.

The observation that HITS was a predictor of IEs in CAD but not in vertebral dissections (VAD) in our subgroup analysis warrants further discussion. This difference could be attributed to several factors. First, the hemodynamics and vessel wall characteristics differ significantly between carotid and vertebral arteries, which can affect the detection and significance of HITS. As highlighted in the studies by Brandt et al and Demchuk et al, there are significant concerns about the reliability of TCD in detecting embolic signals within the posterior circulation.^20^, ^21^ Second, the posterior circulation, including the vertebral arteries, is less accessible, making it more challenging for TCD monitoring to accurately detect HITS in the posterior cerebral or basilar arteries compared with the carotid arteries. It is important to acknowledge that the variation in the technique of HITS detection in VAD studies contributed to heterogeneity (I^2^ = 42%) in our subgroup analysis of VAD whereas there was no heterogeneity in the CAD subgroup (I^2^ = 0%). This highlights the need for future studies to consider convergence and standardization of HITS detection techniques in the posterior circulation to reduce heterogeneity and improve the reliability of results. Novel methods of head‐frame monitoring of the vertebrobasilar junction instead of hand‐held methods may be helpful in detecting HITS in posterior circulation.^15^ Finally, the incidence and pathophysiological mechanisms of IEs may vary between these vascular territories, contributing to the differences observed in predictive values.

Regarding VADs, Perez–Roman et al conducted a retrospective study of 104 patients with vertebral artery dissection, where HITS was detected using TCD performed on posterior cerebral artery territory. Incidence of stroke was 58.6% and the incidence of HITS was 18%, with no significant association between positive HITS and stroke.^14^ Brunser et al conducted a prospective study involving 93 patients with VAD over a 90‐day follow‐up period. Although the presence of HITS was initially identified as a significant factor for recurrent IEs, it did not retain its significance in the multivariate analysis.^12^ This finding underscores the complexity of predicting IEs in VAD patients and suggests that additional factors may significantly influence these outcomes.

On the other hand, studies that were performed in CAD support the predictive significance of HITS in CAD. In the study conducted by Brunser et al, multivariate analysis revealed that the presence of HITS, combined with abnormal Breath‐Holding index, were the only variables that remained associated with IE recurrence during follow‐up.^13^ Molina et al reported that the detection of HITS on serial TCD monitoring may be linked to an increased risk of early ischemic recurrence in patients with acute internal CAD.^13^ Interestingly, our analysis of IEs in 6 patients with VADs and 28 patients with CADs aligns with these findings. Although HITS were significantly associated with IEs in CADs, no such association was observed in VADs.

Numerous studies have explored the predictors of IEs following CeAD. According to Yaghi et al, several risk factors are consistently identified across multiple studies. These include male gender, smoking history, vertebral artery dissection, the presence of multiple dissections or early recurrent dissection, high‐grade stenosis or occlusion, and intraluminal thrombus.^11^ Our univariable analysis highlighted 3 risk factors for future IEs in patients with CeAD: high‐grade luminal stenosis, luminal thrombosis, and the presence of HITS. In the multivariable model, HITS and luminal thrombosis remained statistically significant. Therefore, it is crucial to monitor for HITS and luminal thrombosis in patients CeAD, as they are significant predictors of future IEs. Identifying these risk factors early can enhance risk stratification and guide more effective management strategies to prevent recurrent IEs in this patient population. Our study has several limitations that warrant acknowledgment. First, the use of antiplatelet therapy prior to initiating TCD in some of the published data may have influenced the detection of HITS, introducing potential confounding effects to our results. Second, there was variability in the timing of TCD and duration of follow‐up among the included studies, which might affect the detection rates of HITS and their predictive value. Third, there is a paucity of data regarding the different types of dissections (spontaneous versus traumatic), which limits our ability to generalize the findings across all types of CeAD. Lastly, the overall low number of cases and studies included in our analysis may limit the statistical power and the robustness of our conclusions. Despite these limitations, this study deepens our understanding of how TCD findings can be interpreted and used predictively in the context of CeAD.

In conclusion, TCD remains an invaluable tool for evaluating patients with CeAD and predicting prognosis in high‐risk individuals. This imaging modality plays a crucial role in guiding treatment decisions, especially for patients with CAD where HITS are detected during TCD evaluations. By identifying at‐risk patients early, TCD can help tailor more effective and targeted treatment strategies, ultimately improving patient outcomes.

## Conflicts of Interest Statement

None.

## Sources of Funding

None.

## Disclosures

None.

## Supporting information




**Table S1**. PRISMA checklist.
**Table S2**. Search syntax for all databases.
**Table S3**. STROBE checklist.
**Table S4**. Quality assessment of cohort studies based on NOS.
**Table S5**. Quality of case series based on Murad et al's tool.
**Table S6**. Patient characteristics based on presence or absence of ischemic events within 90 days.
**Table S7**. Univariable and multivariable logistic regression analyses evaluating the association with risk of recurrent ischemic events.
**Table S8**. Patients' characteristics according to the presence or absence of HITS.
