# Supplementary material for: Risk Prediction of Cerebrovascular Ischemic Events Following Cervical Artery Dissections Using High‐Intensity Transient Signals: A Systematic Review, Meta‐Analysis and a Single Center Experience
Source: Stroke Vasc Interv Neurol. 2025 Mar 8;5(3):e001704. doi: 10.1161/SVIN.124.001704 (PMC12180476; doi:10.1161/SVIN.124.001704)
Supplement: Supplementary file 1 — Table S1. PRISMA checklist. Table S2. Search syntax for all databases. Table S3. STROBE checklist. Table S4. Quality assessment of cohort studies based on NOS. Table S5. Quality of case series based on Murad et al's tool. Table S6. Patient characteristics based on presence or absence of ischemic events within 90 days. Table S7. Univariable and multivariable logistic regression analyses evaluating the association with risk of recurrent ischemic events. Table S8. Patients' characteristics according to the presence or absence of HITS. [file SVI2-5-e001704-s001.pdf]

SUPPLEMENTAL MATERIALS

Risk Prediction of Cerebrovascular Ischemic Events Following Cervical Artery Dissections Using High-Intensity Transient Signals: A Systematic Review, Meta-Analysis and a single center experience

Seyed Behnam Jazayeri[1], Behnam Sabayan[2,3], Yasaman Pirahanchi[4], Vikas Ravi[4], Julián Carrión-Penagos[4], Jeffery Bowers[4], Royya Modir[4], Kunal Agrawal[4], Thomas Hemmen[4], Brett Meyer[4], Dawn Meyer[4], Reza Bavarsad Shahripour[4]

[1] Tehran University of Medical Sciences, Tehran, Iran

[2] Department of Neurology, Hennepin Healthcare Research Institute, Minneapolis, Minnesota, USA

[3] Division of Epidemiology and Community Health, School of Public Health, University of Minnesota, Minneapolis, Minnesota, USA

[4] University of California San Diego, Neuroscience Department, Comprehensive Stroke Center, San Diego, California, USA

*Corresponding author: Reza Bavarsad Shahripour, MD, FAHA, RPNI

Content:

Expanded Materials & Methods.

Expanded Results

Table S1. PRISMA checklist

Table S2. Search syntax for all databases

Table S3. STROBE checklist

Table S4. Quality assessment of cohort studies based on NOS

Table S5. Quality of case series based on Murad et al’s tool.

Table S6. Patient characteristics based on presence or absence of ischemic events within 90 days

Table S7. Univariable and multivariable logistic regression analyses evaluating the association with risk of recurrent ischemic events.

Table S8. Patients’ characteristics according to the presence or absence of HITS

Expanded Materials & Methods

Details of our single center study:

Our observational study adhered to guidelines of reporting for observational studies. The Strengthening of the Reporting of Observational Studies in Epidemiology (STROBE) checklist is provided in Table S3[1]. This study was approved by the Ethics Committee of the University of California, San Diego (IRB# 810210). The Ethics Committee at our institution waived the need for written patient consent. TCD is a part of standard care in the management of patients with stroke in our department with no safety issues.

Study population

We conducted a retrospective study involving all consecutive patients with CeAD who were admitted to UC San Diego Medical Center between February 2021 and December 2023. Patients were diagnosed based on the clinical suspicion of a vascular neurologist, and their diagnoses were later confirmed with a neuroradiologist using imaging techniques including CTA (Computed Tomography Angiography), MRA (Magnetic Resonance Angiography), or traditional angiography of the carotid and vertebral arteries[2]. The inclusion criteria for this study were as follows: (1) carotid or vertebral artery dissection confirmed by advanced imaging, (2) age ≥18 years, and (3) TCD study during the first 72h from symptom onset. We did not enroll patients with (1) poor TCD window (2) traumatic dissections and (3) patients with TCD evaluation after 72h from symptoms.

Measures and definitions

The following baseline variables were obtained from our hospital database: age, gender, symptoms at diagnosis, dissected artery side, presence or absence of HITS, luminal thrombosis and degree of luminal stenosis according to the North American Symptomatic Carotid Endarterectomy Trial (NASCET) criteria[3] (mild (<30%), moderate (30–69%), or severe (70–99%). Additionally, the patient's medical history was recorded, including smoking, hypertension (HTN), diabetes mellitus (DM), hyperlipidemia (HLP), migraine, history of minor trauma (intense exercise, Yoga, massage), recent infection or severe cough in the last 7 days before dissection. Patients' antithrombotic or anticoagulation choice during follow-up was also recorded as single antiplatelet therapy (SAPT), dual antiplatelet therapy (DAPT), or anticoagulant (AC) therapy.

Transcranial Doppler was performed as a routine evaluation for all patients with suspected CeAD within the first 24 hours after admission. The procedure was conducted by a highly experienced vascular neurologist with a fellowship in neurosonology (R.B.S.), who has over 15 years of experience in TCD. A DWL TCD machine (Multi-Dop T digital) was used for each patient, with TCD durations varying between 15 to 30 minutes for monitoring the target vessels (Middle Cerebral Artery (MCA) M1 segment at a depth of 55-65 mm or basilar artery at a depth of 75-95 mm). For MCA monitoring, the probe was securely fixed to the patients using a head frame, ensuring a consistent angle for evaluation and minimizing artifacts. Additionally, in cases of vertebral dissection, the basilar arteries were monitored. For our study, we defined CeAD as the presence of intramural hematoma (bleeding within the artery wall), an intimal flap (a tear in the artery's inner layer), a double lumen (the appearance of two channels within the artery), or a pseudoaneurysm (a bulging of the artery wall due to injury)[2].

HITS were identified if the following conditions were met: (1) random occurrence, (2) less than 0.1 seconds, (3) high intensity, at least 3 dB more than background intensity, showing a spike in intensity, (4) unidirectional quality in the Doppler spectrum, and 5) co-occurrence with a “chirp” or “pop” sound [4, 5]. Alongside HITS detection, luminal thrombosis and the degree of stenosis recorded through CTA or MRA were also noted.

All patients included in the study were followed through routine clinic visits or phone call interviews for three months. Recurrent stroke was defined as any neurological deficit lasting more than 24 hours within 90 days of initial admission, with ischemic lesions confirmed by diffusion-weighted imaging (DWI) MRI. TIA was also considered if patients presented with a focal neurological deficit were examined and diagnosed by a vascular neurologist as having a TIA, and showed no evidence of a new stroke on MRI. Patients’ functional outcomes were evaluated using modified Rankin Scale (mRS) at the end of 90 days.

Statistical Analysis

Statistical analyses were performed using SPSS version 16.0. Continuous variables were expressed as mean ± standard deviation (SD) or median with interquartile range (IQR), while categorical variables were presented as counts and proportions. The Shapiro-Wilk test assessed the normal distribution of the variables. Continuous variables with a normal distribution were compared using Student’s t-test or ANOVA, while non-normally

distributed variables were analyzed using the Mann-Whitney U test or Kruskal-Wallis test. Univariable analysis, employing Fisher exact tests and t-tests for categorical and continuous variables, respectively, identified potential predictors of stroke or TIA within 90 days post-CeAD. Variables tested included gender, age, smoking status, migraine, dissected artery (carotid vs vertebral), degree of luminal stenosis, intraluminal thrombus, intraluminal hematoma, multiple or recurrent dissections, vascular surface irregularity, and presence of HITS. Significant factors ($p \leq 0.10$) were included in a multivariable model using a backward stepwise multiple logistic regression. Variables with $p \leq 0.05$ were retained in the multivariable model. Odds ratios (ORs) and 95% confidence intervals (CIs) were calculated for all factors associated with 90-day stroke/TIA recurrence.

Expanded results:

Single center study

A total of 39 patients with CeAD were identified from the hospital registry during the study period. Five patients were excluded due to poor TCD window. The mean age of the patients was 46.8 ± 11.2 years, and 56% were male. All patients underwent CTA, 14 (41.2%) also underwent MRA, and 9 (26.5%) patients underwent angiography. Most of the dissections were right-sided (70.6%) and were located in the carotid artery (82.4%). All included patients are followed until 90 days with no missing data on follow up.

Table S6 provides a summary of the baseline characteristics of patients with and without IEs at follow-up. Seven patients experienced IEs during the 90-day follow-up including 3 TIAs and 4 ischemic strokes. All IEs occurred during the first month post-CeAD including <1 week in 1/7 (14%), 1-2 weeks in 4/7 (57%) and 2-3 weeks in 2/7 (29%) of patients. Common initial CeAD symptoms were neck pain (41%), headache (38%) and pulsatile tinnitus (9%). No patients presented with Horner syndrome. A history of minor trauma (massage, lifting heavy weights, and yoga) was present in 12 patients (35.3%) before the dissection and was more common among patients younger than 45 years old ($p = 0.03$) . Patients with IEs exhibited significantly more severe luminal stenosis at onset ($p < 0.01$) and a higher prevalence of HITS (100% vs. 33%, $p = 0.002$) compared to those without IEs. In addition the proportion of patients with recurrent or multiple CeAD was higher among patients who experienced IE (42.9% vs 7.4%, $p=0.08$).

However, there was no difference in age, gender, dissected artery, laterality, timing of TCD, frequency of smoking or other comorbidities, luminal thrombosis, luminal hematoma, vessel surface irregularity, onset to arrival time, and presenting symptoms between groups with and without IEs.

Table S7 presents the results of both univariable and multivariable analyses. In the univariable analysis, high-grade luminal stenosis, luminal thrombosis, and the presence of HITS were significantly associated with recurrent IEs ($p < 0.1$). However, variables such as gender, smoking history, migraine, dissected artery, recurrent/multiple dissections, vessel surface irregularity, luminal hematoma, and onset to arrival time did not show significant associations with recurrent IEs in the univariable analyses. In the multivariable analysis, the presence of HITS (OR 29.7, 95% CI: 2.5–341.7, p=0.006) and luminal thrombosis (OR 20, 95% CI: 1.4-282, p=0.02) remained significant predictors of IEs, after adjusting for the degree of luminal stenosis.

Table S8 shows patient characteristics based on presence or absence of HITS. In total, HITS were observed in 16 patients (47%). Patients with HITS had similar age, gender, dissected artery, comorbidities, and luminal thrombosis to patients without HITS. However, luminal stenosis was higher in patients with HITS (p=0.008). Among 16 patients with HITS, 7 patients experienced IEs (43.7%) compared with no IEs among 18 patients without HITS. The difference was statistically significant (p=0.002).

Table S1. PRISMA checklist.

Section and Topic	Item #	Checklist item	Location where item is reported
**TITLE**			
Title	1	Identify the report as a systematic review.	1
**ABSTRACT**			
Abstract	2	See the PRISMA 2020 for Abstracts checklist.	Abstract
**INTRODUCTION**			
Rationale	3	Describe the rationale for the review in the context of existing knowledge.	5
Objectives	4	Provide an explicit statement of the objective(s) or question(s) the review addresses.	5
**METHODS**			
Eligibility criteria	5	Specify the inclusion and exclusion criteria for the review and how studies were grouped for the syntheses.	5-6
Information sources	6	Specify all databases, registers, websites, organisations, reference lists and other sources searched or consulted to identify studies. Specify the date when each source was last searched or consulted.	5
Search strategy	7	Present the full search strategies for all databases, registers and websites, including any filters and limits used.	Supp materials
Selection process	8	Specify the methods used to decide whether a study met the inclusion criteria of the review, including how many reviewers screened each record and each report retrieved, whether they worked independently, and if applicable, details of automation tools used in the process.	6
Data collection process	9	Specify the methods used to collect data from reports, including how many reviewers collected data from each report, whether they worked independently, any processes for obtaining or confirming data from study investigators, and if applicable, details of automation tools used in the process.	6
Data items	10a	List and define all outcomes for which data were sought. Specify whether all results that were compatible with each outcome domain in each study were sought (e.g. for all measures, time points, analyses), and if not, the methods used to decide which results to collect.	6

Section and Topic	Item #	Checklist item	Location where item is reported
	10b	List and define all other variables for which data were sought (e.g. participant and intervention characteristics, funding sources). Describe any assumptions made about any missing or unclear information.	6
Study risk of bias assessment	11	Specify the methods used to assess risk of bias in the included studies, including details of the tool(s) used, how many reviewers assessed each study and whether they worked independently, and if applicable, details of automation tools used in the process.	6
Effect measures	12	Specify for each outcome the effect measure(s) (e.g. risk ratio, mean difference) used in the synthesis or presentation of results.	6-7
Synthesis methods	13a	Describe the processes used to decide which studies were eligible for each synthesis (e.g. tabulating the study intervention characteristics and comparing against the planned groups for each synthesis (item #5)).	6-7
	13b	Describe any methods required to prepare the data for presentation or synthesis, such as handling of missing summary statistics, or data conversions.	-
	13c	Describe any methods used to tabulate or visually display results of individual studies and syntheses.	6
	13d	Describe any methods used to synthesize results and provide a rationale for the choice(s). If meta-analysis was performed, describe the model(s), method(s) to identify the presence and extent of statistical heterogeneity, and software package(s) used.	6
	13e	Describe any methods used to explore possible causes of heterogeneity among study results (e.g. subgroup analysis, meta-regression).	6
	13f	Describe any sensitivity analyses conducted to assess robustness of the synthesized results.	6
Reporting bias assessment	14	Describe any methods used to assess risk of bias due to missing results in a synthesis (arising from reporting biases).	N/A
Certainty assessment	15	Describe any methods used to assess certainty (or confidence) in the body of evidence for an outcome.	N/A
**RESULTS**			
Study selection	16a	Describe the results of the search and selection process, from the number of records identified in the search to the number of studies included in the review, ideally using a flow diagram.	7
	16b	Cite studies that might appear to meet the inclusion criteria, but which were excluded, and explain why they were excluded.	7
Study characteristics	17	Cite each included study and present its characteristics.	Page 7 and tables

Section and Topic	Item #	Checklist item	Location where item is reported
Risk of bias in studies	18	Present assessments of risk of bias for each included study.	Supp materials
Results of individual studies	19	For all outcomes, present, for each study: (a) summary statistics for each group (where appropriate) and (b) an effect estimate and its precision (e.g. confidence/credible interval), ideally using structured tables or plots.	Table 1
Results of syntheses	20a	For each synthesis, briefly summarise the characteristics and risk of bias among contributing studies.	Supp materials
	20b	Present results of all statistical syntheses conducted. If meta-analysis was done, present for each the summary estimate and its precision (e.g. confidence/credible interval) and measures of statistical heterogeneity. If comparing groups, describe the direction of the effect.	Figures of meta-analysis
	20c	Present results of all investigations of possible causes of heterogeneity among study results.	Figures of meta-analysis
	20d	Present results of all sensitivity analyses conducted to assess the robustness of the synthesized results.	N/A
Reporting biases	21	Present assessments of risk of bias due to missing results (arising from reporting biases) for each synthesis assessed.	N/A
Certainty of evidence	22	Present assessments of certainty (or confidence) in the body of evidence for each outcome assessed.	N/A
**DISCUSSION**			
Discussion	23a	Provide a general interpretation of the results in the context of other evidence.	8-9
	23b	Discuss any limitations of the evidence included in the review.	10
	23c	Discuss any limitations of the review processes used.	10
	23d	Discuss implications of the results for practice, policy, and future research.	10
**OTHER INFORMATION**			
Registration and protocol	24a	Provide registration information for the review, including register name and registration number, or state that the review was not registered.	5

Section and Topic	Item #	Checklist item	Location where item is reported
	24b	Indicate where the review protocol can be accessed, or state that a protocol was not prepared.	5
	24c	Describe and explain any amendments to information provided at registration or in the protocol.	N/A
Support	25	Describe sources of financial or non-financial support for the review, and the role of the funders or sponsors in the review.	11
Competing interests	26	Declare any competing interests of review authors.	11
Availability of data, code and other materials	27	Report which of the following are publicly available and where they can be found: template data collection forms; data extracted from included studies; data used for all analyses; analytic code; any other materials used in the review.	11

From: Page MJ, McKenzie JE, Bossuyt PM, Boutron I, Hoffmann TC, Mulrow CD, et al. The PRISMA 2020 statement: an updated guideline for reporting systematic reviews. BMJ 2021;372:n71. doi: 10.1136/bmj.n71

Table S2. Search syntax for all databases

**Date of search: 2/9/2024**
**Pubmed: 120**
((((cervical artery dissection) OR (carotid artery dissection) OR (vertebral artery dissection))) AND (((transcranial ultrasound) OR (transcranial doppler) OR (transcranial sono*) OR (HITS) OR (micro*embol*))))) AND ((stroke) OR (TIA))
**Embase: 229**
(cervical AND artery AND dissection OR (carotid AND artery AND dissection) OR (vertebral AND artery AND dissection)) AND (transcranial AND ultrasound OR (transcranial AND doppler) OR (transcranial AND sono*) OR hits OR micro*embol*) AND ('stroke'/exp OR stroke OR 'transient ischemic attack'/exp OR 'transient ischemic attack')
**Scopus: 156**
TITLE-ABS-KEY ((('cervical AND artery AND dissection') OR ('carotid AND artery AND dissection') OR ('vertebral AND artery AND dissection')) AND (('transcranial AND ultrasound') OR ('transcranial AND doppler') OR ('transcranial AND sono*') OR ('hits') OR ('microembol*')) AND ('stroke' OR 'tia'))

Table S3. STROBE Statement—checklist of items that should be included in reports of observational studies

	Item No	Recommendation	Page No
**Title and abstract**	1	(*a*) Indicate the study's design with a commonly used term in the title or the abstract	3
		(*b*) Provide in the abstract an informative and balanced summary of what was done and what was found	3
**Introduction**			
Background/rationale	2	Explain the scientific background and rationale for the investigation being reported	5
Objectives	3	State specific objectives, including any prespecified hypotheses	5
**Methods**			
Study design	4	Present key elements of study design early in the paper	7
Setting	5	Describe the setting, locations, and relevant dates, including periods of recruitment, exposure, follow-up, and data collection	Supp materials
Participants	6	(*a*) *Cohort study*—Give the eligibility criteria, and the sources and methods of selection of participants. Describe methods of follow-up<br>*Case-control study*—Give the eligibility criteria, and the sources and methods of case ascertainment and control selection. Give the rationale for the choice of cases and controls<br>*Cross-sectional study*—Give the eligibility criteria, and the sources and methods of selection of participants	Supp materials
		(*b*) *Cohort study*—For matched studies, give matching criteria and number of exposed and unexposed<br>*Case-control study*—For matched studies, give matching criteria and the number of controls per case	Table S6 and S7
Variables	7	Clearly define all outcomes, exposures, predictors, potential confounders, and effect modifiers. Give diagnostic criteria, if applicable	Supp materials

Data sources/ measurement	8*	For each variable of interest, give sources of data and details of methods of assessment (measurement). Describe comparability of assessment methods if there is more than one group	Supp materials
Bias	9	Describe any efforts to address potential sources of bias	Supp materials
Study size	10	Explain how the study size was arrived at	Supp materials
Quantitative variables	11	Explain how quantitative variables were handled in the analyses. If applicable, describe which groupings were chosen and why	Supp materials
Statistical methods	12	(*a*) Describe all statistical methods, including those used to control for confounding	Supp materials
		(*b*) Describe any methods used to examine subgroups and interactions	Supp materials
		(*c*) Explain how missing data were addressed	N/A
		(*d*) *Cohort study*—If applicable, explain how loss to follow-up was addressed<br>*Case-control study*—If applicable, explain how matching of cases and controls was addressed<br>*Cross-sectional study*—If applicable, describe analytical methods taking account of sampling strategy	N/A
		(*e*) Describe any sensitivity analyses	N/A

Continued on next page

Results

Participants	13*	(a) Report numbers of individuals at each stage of study—eg numbers potentially eligible, examined for eligibility, confirmed eligible, included in the study, completing follow-up, and analysed	Supp materials
		(b) Give reasons for non-participation at each stage	Supp materials
		(c) Consider use of a flow diagram	N/A
Descriptive data	14*	(a) Give characteristics of study participants (eg demographic, clinical, social) and information on exposures and potential confounders	Supp materials
		(b) Indicate number of participants with missing data for each variable of interest	N/A
		(c) *Cohort study*—Summarise follow-up time (eg, average and total amount)	Supp materials
Outcome data	15*	*Cohort study*—Report numbers of outcome events or summary measures over time	*7-8*
		Case-control study—Report numbers in each exposure category, or summary measures of exposure	*N/A*
		Cross-sectional study—Report numbers of outcome events or summary measures	*N/A*
Main results	16	(*a*) Give unadjusted estimates and, if applicable, confounder-adjusted estimates and their precision (eg, 95% confidence interval). Make clear which confounders were adjusted for and why they were included	8
		(*b*) Report category boundaries when continuous variables were categorized	N/A

		(*c*) If relevant, consider translating estimates of relative risk into absolute risk for a meaningful time period	N/A
Other analyses	17	Report other analyses done—eg analyses of subgroups and interactions, and sensitivity analyses	N/A
**Discussion**			
Key results	18	Summarise key results with reference to study objectives	8
Limitations	19	Discuss limitations of the study, taking into account sources of potential bias or imprecision. Discuss both direction and magnitude of any potential bias	10
Interpretation	20	Give a cautious overall interpretation of results considering objectives, limitations, multiplicity of analyses, results from similar studies, and other relevant evidence	9-10
Generalisability	21	Discuss the generalisability (external validity) of the study results	10
**Other information**			
Funding	22	Give the source of funding and the role of the funders for the present study and, if applicable, for the original study on which the present article is based	11

Table S4. Quality assessment of cohort studies based on NOS

Signaling questions	Brunser, 2016	Brunser, 2020	Molina, 2000	Perez-Roman, 2024	Jazayeri, 2024
SELECTION					
*1) Representativeness of the Exposed Cohort	*	*	*	*	*
*2) Selection of the Non-Exposed Cohort	*	*	*	*	*
*3) Ascertainment of Exposure	*	*	*	*	*
*4) Demonstration That Outcome of Interest Was Not Present at Start of Study	*	*	*	*	*
COMPARABILITY					
**1) Comparability of Cohorts on the Basis of the Design or Analysis	**	**	-	**	**
OUTCOME					
*1) Assessment of Outcome	*	*	*	*	*
*2) Was Follow-Up Long Enough for Outcomes to Occur	*	*	-	*	*
*3) Adequacy of Follow Up of Cohorts	*	*	-	*	*
Total score	9	9	5	9	9
Interpretation	Low risk	Low risk	Some concerns	Low risk	Low risk

Table S5. Quality of case series based on Murad et al's tool.

Domains	Leading explanatory questions (Signaling questions)	Yamaoka, 2014
Selection	1. Does the patient(s) represent(s) the whole experience of the investigator (centre) or is the selection method unclear to the extent that other patients with similar presentation may not have been reported?	Yes
Ascertainment	2. Was the exposure adequately ascertained?	Yes
Ascertainment	3. Was the outcome adequately ascertained?	Yes
Causality	4. Were other alternative causes that may explain the observation ruled out?	-
Causality	5. Was there a challenge/rechallenge phenomenon?	-
Causality	6. Was there a dose–response effect?	-
Causality	7. Was follow-up long enough for outcomes to occur?	Unclear
Reporting	8. Is the case(s) described with sufficient details to allow other investigators to replicate the research or to allow practitioners make inferences related to their own practice?	Yes
Interpretation		Some concerns

*Questions 4, 5 and 6 are mostly relevant to cases of adverse drug events and were not scored. Therefore, the maximum total possible score was 5.

Table S6. Patient Characteristics Based on Presence or Absence of Ischemic Events Within 90 Days

Variable		Total	No TIA or Stroke within 90 days (n=27)	Recurrent TIA or stroke within 90 days (n=7)	P value
**Age, mean (SD)**		46.8 (11.2)	48.5 (11.3)	40.1 (8.4)	0.07
**CTA**		34 (100)	27 (100)	7 (100)	-
**MRA**		14 (41.2)	10 (37)	4 (57.1)	0.41
**Angiography**		9 (26.5)	6 (22.2)	3 (42.9)	0.34
**Gender**	**Female**	15 (44.1)	10 (37)	2(71.4)	0.19
	**Male**	19 (55.9)	17 (63)	2 (28.6)	
**Laterality**	**Left**	10 (29.4)	8 (29.6)	2 (28.6)	1
	**Right**	24 (70.6)	19 (70.4)	5 (71.4)	
**Dissected artery**	**Vertebral**	6 (17.6)	5 (18.5)	1 (14.3)	0.64
	**Carotid**	28 (82.4)	22 (81.5)	6 (85.7)	
**Time of TCD**	**<24h**	24 (70.6)	20 (74.1)	4 (57.1)	0.5
	**24-48h**	9 (26.5)	6 (22.2)	3 (42.9)	
	**48-72h**	1 (2.9)	1 (3.7)	0	
**Luminal stenosis at dissection diagnosis**	**Mild**	18 (52.9)	18 (66.7)	0 (0.0)	<0.001
	**Moderate**	6 (17.6)	4 (14.8)	2 (28.6)	
	**Severe**	10 (29.4)	5 (18.5)	5 (71.4)	
**Luminal thrombosis**		10 (29.4)	5 (18.5)	5 (71.4)	0.14
**Comorbidities**	**Smoking**	10 (29.4)	7 (25.9)	3 (42.9)	0.39
	**Diabetes Mellitus**	4 (11.8)	4 (14.8)	0	0.55
	**Hypertension**	9 (26.5)	8 (29.6)	1 (14.3)	0.64
	**Hyperlipidemia**	4 (11.8)	4 (14.8)	0	0.55
	**Migraine**	9 (26.5)	7 (25.9)	2 (28.6)	1
**History of OCP use**		3 (8.8)	3 (11.1)	0 (0.0)	1
**Drug abuse**		4 (11.8)	3 (11.1)	1 (14.3)	1
**Alcohol abuse**		3 (8.8)	2 (7.4)	1 (14.3)	1
**Recent infection**		6 (17.6)	4(16.7)	2(20)	1
**Antiplatelet or Anticoagulant use**	**SAPT (ASA)**	15 (44.1)	11 (40.7)	4 (57.1)	0.71
	**DAPT**	17 (50)	13 (48.1)	4 (57.1)	1
	**AC**	7 (20.6)	4 (14.8)	3 (42.9)	0.13
**Diagnosis symptom**	**Neck pain**	14 (41.2)	11 (40.7)	3 (42.9)	1
	**Headache**	13 (38.2)	10 (37)	3 (42.9)	1
	**Pulsatile tinnitus**	3 (8.8)	3 (11.1)	0	1
**NIHSS score at baseline**		2.4 (1.4)	2.5 (1.5)	2.3 (1.4)	0.67

**Onset to arrival time (hours), median (IQR)**	6.5 (4-10.25)	6.5 (4-10.75)	6.5 (3.75-9)	0.65
**Intraluminal hematoma**	4 (11.7)	2 (7.4)	2 (28.6)	0.18
**Vessel surface irregularity**	5 (14.7)	4 (14.8)	1 (4.3)	1
**Multiple or recurrent dissections**	5 (14.7)	2 (7.4)	3 (42.9)	0.048
**Presence of HITS on TCD**	16 (47)	9 (33.3)	7 (100)	0.002
**mRS 0-1 at 3 months**	29 (85.3)	23 (85.2)	6 (85.7)	1
TIA: Transient Ischemic Attack; CTA: Computed Tomography Angiography; MRA: Magnetic Resonance Angiography; TCD: Transcranial Doppler; OCP: Oral Contraceptive Pill; SAPT: Single Antiplatelet Therapy; ASA: Aspirin; DAPT: Dual Antiplatelet Therapy; AC: Anticoagulant; NIHSS: National Institutes of Health Stroke Scale; HITS: high-intensity transient signals; mRS: modified Rankin Scale				

Table S7: Univariable and Multivariable Logistic Regression Analyses Evaluating the Association with Risk of Recurrent IEs.

Effect	OR	95% CI	p-value
**Sex (male vs female)**	0.2	0.04-1.4	0.11
**Age (>45 vs <45y)**	0.2	0.04-1.6	0.16
**Smoking (yes vs no)**	2.1	0.38-12.0	0.38
**Migraine (yes vs no)**	1.1	0.17-7.2	0.88
**Dissected artery (carotid vs vertebral)**	0.7	0.07-7.5	0.79
**Degree of luminal stenosis (severe vs mild-to-moderate)**	6.4	1.5-26.2	0.009*
**Luminal thrombosis (yes vs no)**	11	1.6-74	0.01*
**Luminal hematoma (yes vs no)**	5	0.5-44.3	0.14
**Vessel surface irregularity (yes vs no)**	0.95	0.09-10.2	0.97
**Multiple or recurrent dissections (yes vs no)**	4.3	0.23-79.5	0.32
**HITS (yes vs no)**	21.8	2.3-206.4	0.007*
**Multivariable analysis**			
**Degree of luminal stenosis (severe vs mild-to-moderate)**	2.3	0.36-15.7	0.7
**Luminal thrombosis (yes vs no)**	20	1.4-282	0.02*
**HITS (yes vs no)**	29.7	2.5-341.7	0.006*

HITS: High-intensity transient signal. *: p value< 0.1 in univariable analysis and <0.05 in multivariable analysis

**Table S8. Patients' characteristics according to the presence or absence of HITS**				
**Variable**	N	HITS - (n=18)	HITS + (n=16)	P value
**Age > 45**	18	10 (55.5)	8 (50)	0.7
**Gender, male**	19	12 (66.6)	7 (43.7)	0.1
**Dissected vessel, carotid**	28	15 (83.3)	13 (81.2)	0.8
**Comorbidities**				
**Smoking**	10	6 (33.3)	4 (25)	0.5
**Hyperlipidemia**	10	6 (33.3)	4 (25)	0.5
**Diabetes mellitus**	4	3 (16.7)	1 (6.2)	0.6
**Hypertension**	9	6 (3.3)	3 (18.7)	0.3
**Migraine**	9	5 (27.7)	4 (25)	0.8
**Drug abuse**	4	2 (11.1)	2 (12.5)	0.9
**Luminal thrombus**	10	4 (22.2)	6 (37.5)	0.3
**Dissection side, right**	24	13 (72.2)	11 (68.7)	0.8
**Luminal stenosis degree at baseline**				
**Mild**	18	14 (77.7)	4 (25)	0.008
**Moderate**	6	2 (11.1)	4 (25)	
**Severe**	10	2 (11.1)	8 (50)	
**Outcomes**				
**Recurrent TIA/Stroke**	10	0 (0)	7 (43.7)	0.002
**mRS 0-1 at 3 months**	29	15 (83.3)	14 (87.5)	1
HITS: High-intensity transient signal; TIA: Transient Ischemic Attack; mRS: modified Rankin Scale				

References used in supplemental materials.

1. von Elm E, Altman DG, Egger M, Pocock SJ, Gøtzsche PC, Vandenbroucke JP. The Strengthening the Reporting of Observational Studies in Epidemiology (STROBE) statement: guidelines for reporting observational studies. *Ann Intern Med*. Oct 16 2007;147(8):573-7. doi:10.7326/0003-4819-147-8-200710160-00010
2. Debette S, Leys D. Cervical-artery dissections: predisposing factors, diagnosis, and outcome. *The Lancet Neurology*. 2009;8(7):668-678.
3. Ferguson GG, Eliasziw M, Barr HW, et al. The North American Symptomatic Carotid Endarterectomy Trial : surgical results in 1415 patients. *Stroke*. Sep 1999;30(9):1751-8. doi:10.1161/01.str.30.9.1751
4. Gilad R, Lampl Y, Eschel Y, Sadeh M. Antiepileptic treatment in patients with early postischemic stroke seizures: a retrospective study. *Cerebrovascular Diseases*. 2001;12(1):39-43.
5. Markus H, Droste DW, Brown MM. Detection of asymptomatic cerebral embolic signals with Doppler ultrasound. *The Lancet*. 1994;343(8904):1011-1012.
